# Evaluation of 0 ≤ *M* ≤ 8 earthquake data sets in African – Asian region during 1966–2015

**DOI:** 10.1016/j.dib.2018.01.049

**Published:** 2018-01-31

**Authors:** Theophilus Aanuoluwa Adagunodo, Sebastian Lüning, Adekunle Michael Adeleke, Julius Oluwasegun Omidiora, Ahzegbobor Philips Aizebeokhai, Kehinde David Oyeyemi, Olaide Sakiru Hammed

**Affiliations:** a Department of Physics, Covenant University, Ota, Ogun State, Nigeria; b Institute for Hydrography, Geoecology and Climate Sciences, Hauptstraße 47, 6315 Ägeri, Switzerland; c Department of Physics, University of Ilorin, Ilorin, Kwara State, Nigeria; d Department of Languages and General Studies, Covenant University, Ota, Ogun State, Nigeria; e Department of Physics, Federal University of Oye Ekiti, Oye Ekiti, Ekiti State, Nigeria

**Keywords:** African plates, Arabian plates, *b*-value, *Gutenberg-Richter’s model*, Artificial neural network, Earthquake magnitudes, Focal depth, Seismic events, Seismographs, Time series, Tectonic stress

## Abstract

This article evaluates the occurrence of 0 ≤*M*≤ 8 earthquake data sets for the period of 50 years (that is, January 1, 1966 to December 31, 2015) in African and Western Asia region. It is bounded by latitude 40° S to 40° N and longitude 30° W to 60° E with the focal depth of 0–700 km. Seventy seven thousand, six hundred and ninety-six data points were presented for the analysis. The data used were extracted from earthquake catalog of Advanced National Seismic system via http://quake.geo.berkeley.edu/cnss/, an official website of the Northern California Earthquake Data Centre, USA. Each datum comprised the earthquake occurrence date, time of the earthquake occurrence, epicenter’s coordinates, focal depth and magnitude. The Gutenberg-Richter’s relationship being the longest observed empirical relationship in seismology, analysis of variance and time series were used to analyze the seismicity of the study area. Annual distributions of earthquake occurrence based on magnitude variations with the limit 0 ≤*M*≤ 8 were presented. The two constants a and b in the Gutenberg-Richter’s equation, magnitude of completeness (MC) adjusted R-Square and *F*-value for the period of 1966–1975, 1976–1985, 1986–1995, 1996–2005, 2006–2015, and the entire period of investigation ranging from 1966 to 2015 were determined so as to investigate the variations of these parameters on earthquake occurrence over time. The histograms of earthquake occurrence against magnitude of earthquakes for the selected years (1966–1975, 1976–1985, 1986–1995, 1996–2005, 2006–2015, and 1966–2015), and the decadal frequency distributions of earthquake occurrence were also plotted. The focal depth occurrence for each magnitude bins (0–0.9, 1–1.9, 2–2.9, 3–3.9, 4–4.9, 5–5.9, 6–6.9, 7–7.9, 8–8.9) were grouped into shallow, intermediate, and deep depths ranging from 0 to 70, 71 to 300, and 301 to 700 km as being used in seismology. The neural network analysis was also applied to the magnitude of the earthquake. The network uses a time series magnitude data as input with the output being the magnitude of the following day. If the nature of the earthquakes time series is stochastic, modeling and prediction is possible. The earthquake data sets presented in this article can further be adopted in the study of seismicity pattern, *b*-value using series of models, earthquake prediction and variations of earthquake parameters on African and/or Arabian plates. When this approach is integrated with other technique(s), it can provide insights to stability of African lithospehric plates especially the coastal region of Africa.

**Specifications Table**

**Table:** 

Subject area	*Computational Geophysics*
More specific subject area	*Earthquake*
Type of data	*Table and figure*
How data was acquired	*The seismic events were recorded by the seismographs of the Northern California Earthquake Data Centre, USA.*
Data format	*Raw and processed*
Experimental factors	*The data were extracted from the earthquake catalog of Advanced National Seismic system.*
Experimental features	*Computational analysis of earthquake parameters for the period of 50 years (1966–2015) using Microsoft Excel, SPSS and MATLAB R2013a software.*
Data source location	*The data were obtained for 0*≤*M*≤*8 earthquake latitude 40° S to 40° N and longitude 30° W to 60° E, focal depth distribution from 0 to 700km for the period of January 1, 1966 to December 31, 2015. There were 77,696 data points in all.*
Data accessibility	*The data sets are with this article. It is also available on*http://quake.geo.berkeley.edu/cnss/.

**Value of the data**

• Can be used to study the seismicity pattern in African and/or Western Asia region.
• Can be used for *b*-value estimation using integrated models in African – Western Asia seismology.
• Can be used to study the effect of earthquake occurrence on African and/or Arabian lithospheric plates.
• Can be used to estimate the time scale dependence of earthquake parameters in subregions of Africa (Northern, Central, Western, Southern and Eastern Africa) (Fig. 1) and Middle East.

**Fig. 1:**
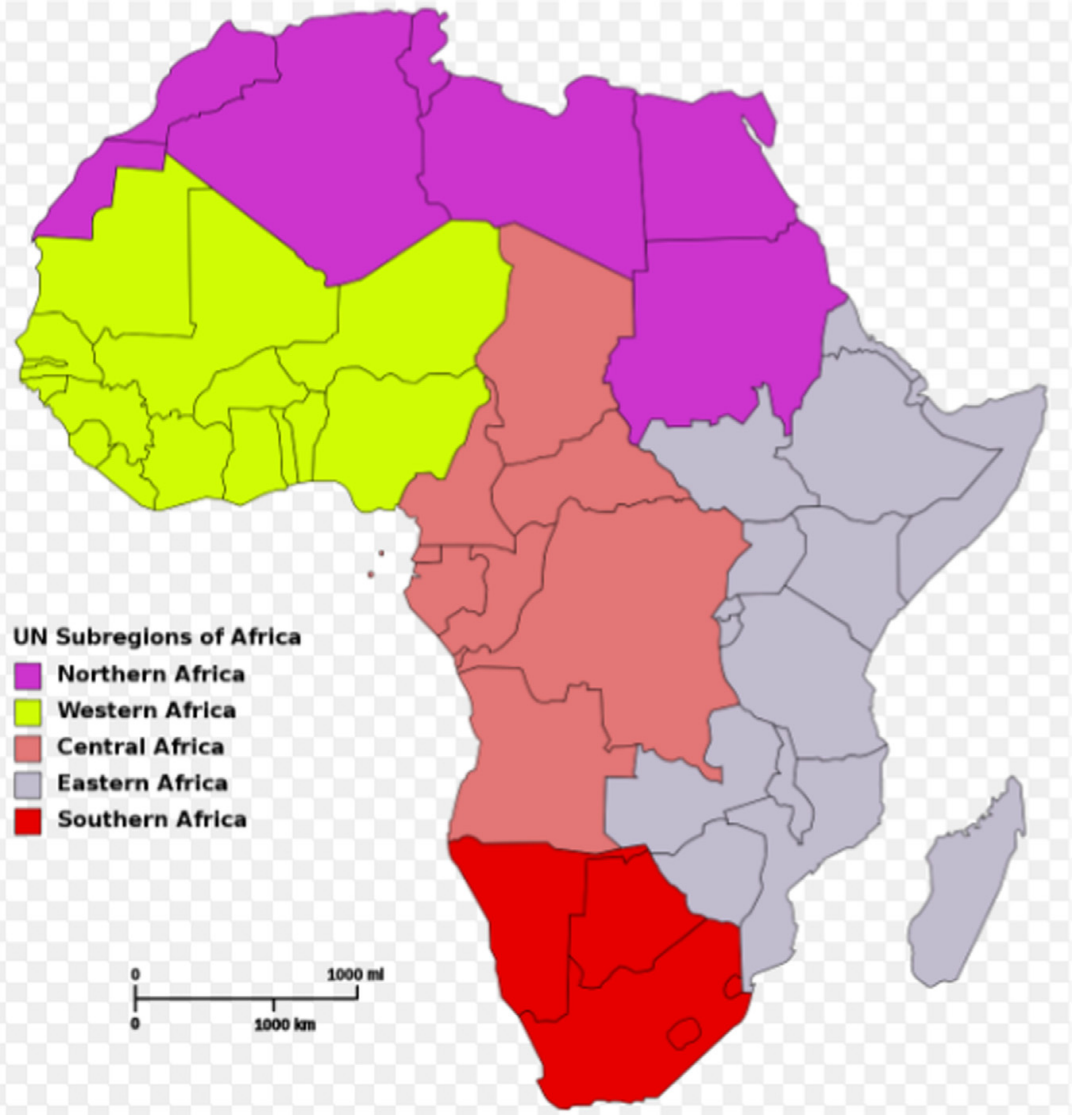
Map of Africa showing her five subregions.
• Can be used to forecast the earthquake occurrence in African and/or Western Asia region.
• Can be integrated with other computational approach for earthquake interpretation.
• Can be used to further explain the stability of African lithospheric plates.
• For educational purposes on seismically active zones in African – Asian region.
• Can be correlated with other earthquake data for seismic activity studies in coastal region of Africa and Middle East.
• Can be employed in the study of seismic activities around the equator when integrated with other techniques such as aeromagnetic data and geographic information system approach.
• It can provide insights to further exploration of aseismic zones being affected by tremors in Africa especially Nigeria.

## 1 Data

**Fig. 2:**
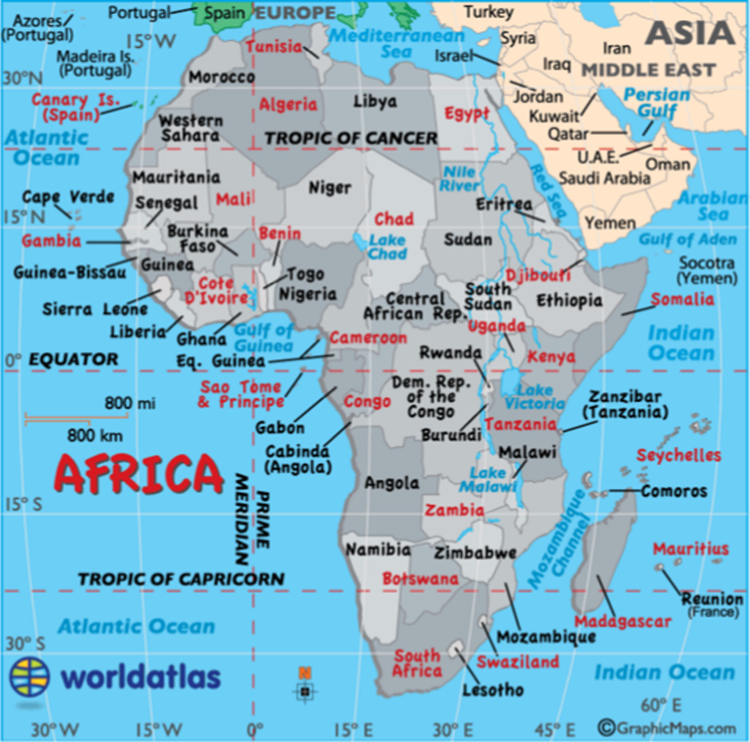
The map of Africa and Asia showing the coverage of the dataset.

The data in this article contains the record of earthquake occurrence in African – Western Asia region. The seismic events were recorded by the seismographs of the Northern California Earthquake Data Centre, USA. The data were obtained for the 0 ≤ *M* ≤ 8 magnitude between latitude 40° S to 40° N and longitude 30° W to 60° E (Fig. 2), focal depth distribution from 0 to 700 km for the period of January 1, 1966 to December 31, 2015. There were 77, 696 data points in all. Each datum comprised the earthquake occurrence date, time of the earthquake occurrence, epicenter’s coordinates, focal depth and magnitude.

An earthquake is caused by a sudden slip along a fault zone. It has been recognized as one of the most destructive of all natural hazards which can severely destroy the entire vicinity in seconds without an explicit warning [1], [2], [3]. Evaluation of earthquake parameters such as magnitude, focal depth and frequency is the fundamental in the study of earthquake pattern and its prediction. These parameters are essential in seismology and serve as reference point to the applied theoreticians. In the study of earthquake, one of the most determined parameters is *b*-value which varied from 0.2 to 2.0, and generally found around 1. This is the measure of stress on the lithospheric plates, because lower *b*-values indicate that the stress is optimum in the investigated region. Generally, very low *b*-values are found in case of immediate aftershocks and higher values are found in case of swarm. There are two mostly used methods in estimation of *b*-values: least square and maximum likelihood methods [4]. In this data article, least square method which is based on Gutenberg-Richter’s (GR) relationship has been adopted to evaluate the data sets of 0 ≤ *M* ≤ 8 magnitude in African – Arabian region to determine the decadal variations of seismicity levels (*a*-values) and tectonic character (*b*-values) for the period of 50 years (1966–2015). Globally, GR equation has been applied to earthquake data for the estimation of *b*-values and related parameters, but few reports from African continent are available in the literature [5] – a gap that is essential to be bridged in the study of seismic activities in Africa and Western Asia region.

The GR law has remained one of the oldest empirical relationships that are still relevant in seismology till date. The relationship is based on power scaling relationship, which relates the frequency and the magnitude of earthquake together in order to predict the degree of stress on the lithospheric plate in a region. The GR equation [6] is presented in Eq. (1).

(1) $$Log_{10}N=a–bM$$

where *N* is the cumulative number of earthquakes of magnitude ≥ *M*, a characterizes the seismicity level of a region, which represents the *M* > 0 earthquake. *b* defines the tectonic character, which is a function of the accumulated stress of a region. In addition, *a* and *b* are constants that vary in space and time.

The descriptive analysis has also been found useful in the evaluation of earthquake occurrence in a region. This ranged from description of earthquake occurrence by plotting the graphs of frequencies of earthquakes against their coordinates [5], number of earthquakes against its magnitudes [7], [8], cumulative number of earthquake against its magnitude [7], and measure of central tendencies [9]. It has been reported that study of previous and present activities of earthquake pattern is vital in prevention of lives and properties from earthquake destructions [9].

Furthermore, in earthquake predictions, several phenomena have been considered by researchers. The considered parameters are electromagnetic fields, seismicity pattern, unusual cloud and weather parameters, unusual emanation of hydrogen and radon gases from the subsurface (e.g. groundwater or soil), animal behaviours [10], and unbalancing level in surface and groundwater [3]. The most unsolved issues in seismology, that is, the time, location and magnitude of the impending earthquake are the major aim of earthquake prediction which can further be improved on via the approach presented at the latter part of this data article.

The neural network developed in this article uses only time series magnitude data as input with the output being the magnitude of the following day. Time series is defined as a sequence of values documented in chronological order over time. Occurrence of previous events may be extremely valuable in prediction of its behaviours in the future. As reported by [11] that, ‘if given a set of past values, it is not possible to predict future values with reliability, the time series is said to be chaotic’. However, if the nature of the earthquakes time series is stochastic, modeling and prediction is possible. The available data in this article can be adapted by the seismologists in understanding, modeling and prediction of earthquake occurrence in African – Arabian region. Furthermore, this analysis can be integrated with other computational approach for better earthquake interpretation. Similar computational analyses to solve other challenges in Man’s environment have been presented in [12], [13], [14].

## 2 Study area, Tectonic Settings and its Geology

**Fig. 3:**
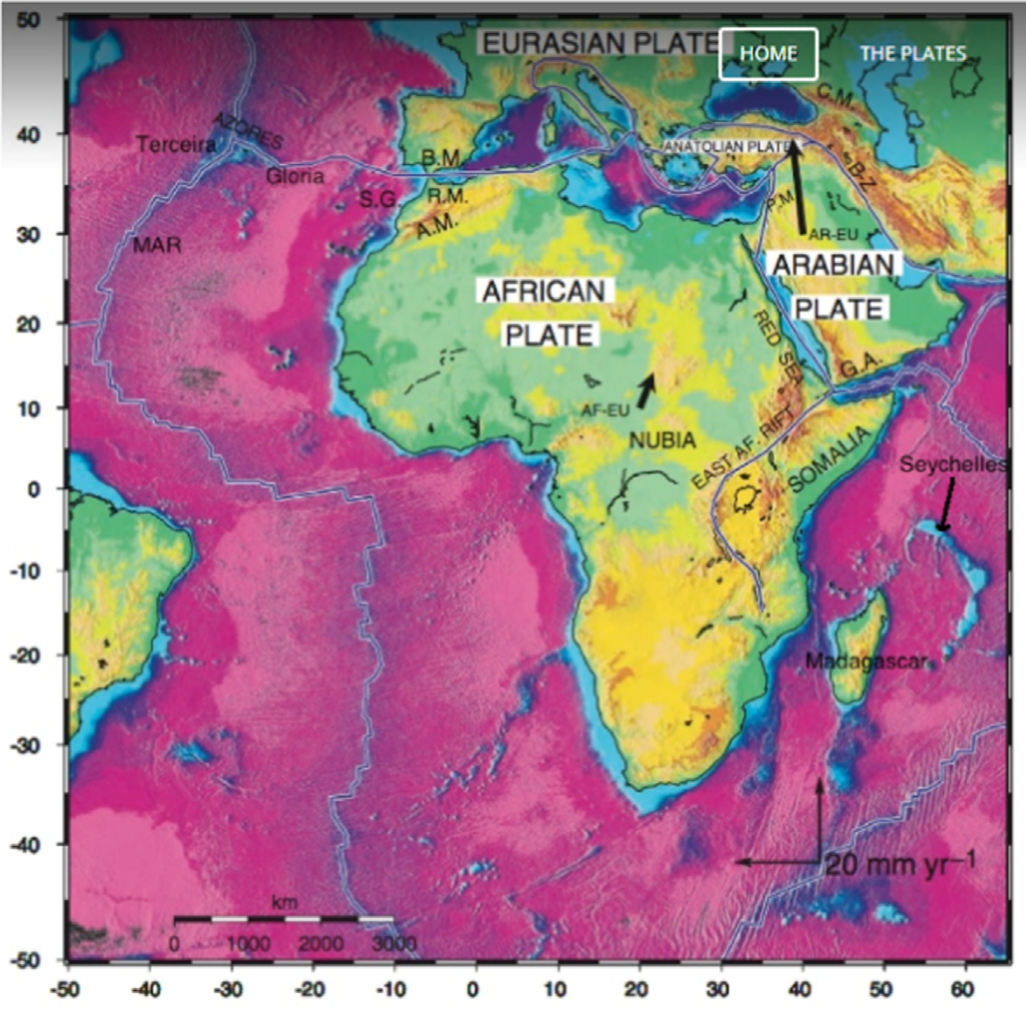
African-Arabian tectonic plates Adapted from [17].

African-Arabian or Western Asia region constitutes all the countries presented in Fig. 2. The study area is bounded by latitude 40° S to 40° N and longitude 30° W to 60° E. The African plate has recently been reported as the third largest plate [5]. It is bounded by a total area of about 60 million square kilometer, with about half of it being covered by land. African plate is composed of old Cratonic units and growth of younger Crust, which represent a period > 2.5 billion years of oceanic and continental crust growth [15]. The African plate is a significant tectonic plate bestriding the equator and the prime meridian. It encapsulates larger percent of the African continent, as well as oceanic Crust which reclines between series of oceanic and continental ridges. The Arabian plate is a minor tectonic plate that falls on the eastern and northern hemispheres. It is one of the three continental plates (the Arabian, Indian, and African plates) that have been moving northward in the recent geological record, and colliding with the Eurasian plate. The African-Arabian region is composed of five tectonic plates: Madagascar, Arabia, Seychelles, Nubia and Somali as presented in Fig. 3. The historical record showed that African tectonic setting was constituted by the breakup of Gondwana in 200 Ma (Mega-annum). This resulted to the interaction of Nubia with Eurasia along the former northern margin [16]. During 160–117 Ma, Madagascar separated from southeastern Africa and rifted to its present location. During the Oligocene (that is, between Eocene and Miocene), the Neotethys Sea (previously located between Nubia and Eurasia) closed through subduction as the two plate collided [17]. The Arabian plate got separated from Africa about 25 Ma ago. This separation led to the closure of the Neotethys Sea, with the succeeding rifting which lead to the formation of Red Sea. From 10 to 60 Ma, the Somali plate began to rift of from African plate. Logatchev et al. [18] predicted another Sea and a new continent between Somali and Africa in the next 1 and 10 Ma respectively. Madagascar and Seychelles (Plateau) plates are microplates within the Somali plate. About 84 Ma, a spreading ridge formed a new location in the Indian Ocean, from which the Mascarene Basin was formed. Further rifting between Seychelles and India at the Tertiary or Cretaceous boundary resulted to the hot spot magmatism, which further sedimented to produce the carbonate shelves on the microplate [19].

**Fig. 4:**
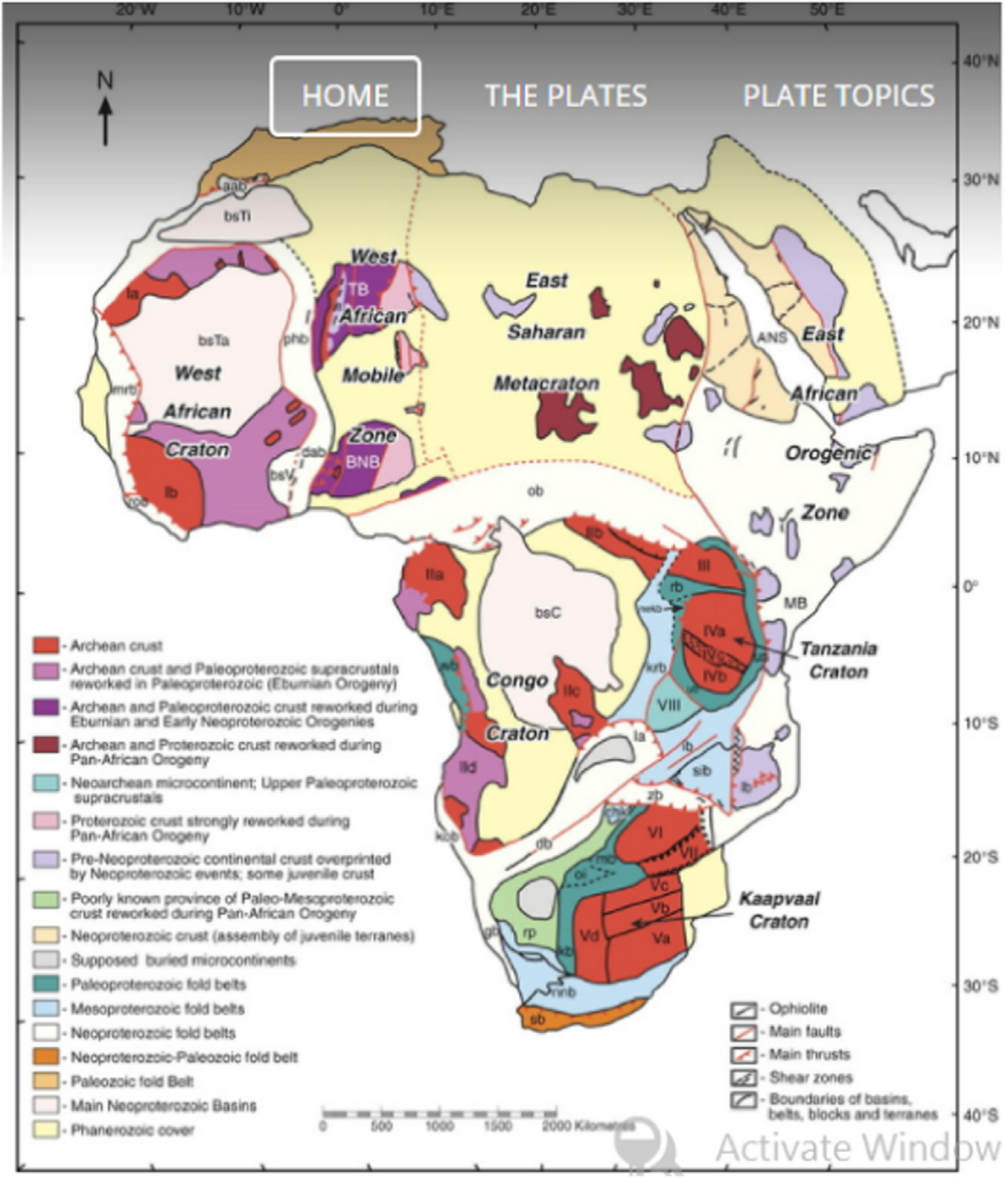
Geological map of Africa.

Generally, Arabian and African continent are made up of a PreCambrian basement of crystalline meta-sedimentary, igneous and meta-igneous rocks (Fig. 4). This crystalline basement is overlain by series of geological settings ranging from volcanic and sedimentary sequences to unconsolidated Cenozoic sediments [20]. African continent is made up of primary units known as Cratons, which are the aforementioned sediments or weathered rocks overlying the crystalline basement.These Cratons are predominantly granitic series, gneisses, and low-grade greenstone belts [20].

## 3 Experimental design, materials and methods

The magnitude of an earthquake is determined based on the information received by the seismograph. The Richter magnitude involves measuring the amplitude of the largest recorded wave at a specific distance from the seismic source. The magnitude of earthquake and its implications are presented in Table 1.

**Table 1:** The Richter magnitude and its implications.

**Magnitude**	**Implications**
0–1	Cannot be felt, but it is detectable by seismograph
2	Smallest quake to be felt. Hangling objects may swing
3	People near the epicenter feel the quake. Comparable to vibrations of a passing truck
4	Causes damage around the epicenter. It is the same as a small fission bomb
5	The weak buildings around the epicenter are damaged
6	Causes greater damage around the epicenter
7	Causes serious damage. Capable to create energy that will heat up a country. It can be felt globally
8	Causes major destruction and death.
9	Rare, but can cause unbelievable damage or total destruction

The annual distributions of seismic activities based on the magnitude of earthquakes are presented in Table 2. The 0 ≤ *M* ≤ 0.9 earthquakes showed total events of 154, 1 ≤ *M* ≤ 1.9 earthquakes showed total events of 2347, 2 ≤ *M* ≤ 2.9 earthquakes showed total events of 17640, 3 ≤ *M* ≤ 3.9 earthquakes showed total events of 33010, 4 ≤ *M* ≤ 4.9 earthquakes showed total events of 20922, 5 ≤ *M* ≤ 5.9 earthquakes showed total events of 3388, 6 ≤ *M* ≤ 6.9 earthquakes showed total events of 216, 7 ≤ *M* ≤ 7.9 earthquakes showed total events of 18, and 8 ≤ *M* ≤ 8.9 earthquakes being the least recorded event occurred once in 1969. Table 2 revealed that African-Arabian magnitude of earthquakes fluctuates between 3 ≤ *M* ≤ 5.9. Seismic events were recorded yearly within this range, which varied from as low as 2 events in 2012 for 3 ≤ *M* ≤ 3.9 to very high events of 3314 in 2008 for the same range of magnitude.

**Table 2:** Yearly distribution of seismic activities according to the magnitude of earthquakes.

**Year/Mag.**	**0–0.9**	**1–1.9**	**2–2.9**	**3–3.9**	**4–4.9**	**5–5.9**	**6–6.9**	**7–7.9**	**8–8.9**
1966	0	0	0	9	230	64	4	0	0
1967	0	0	0	5	179	80	5	0	0
1968	0	1	0	13	209	75	4	1	0
1969	0	0	0	9	241	83	9	0	1
1970	0	0	0	3	268	64	3	1	0
1971	0	0	0	6	223	59	3	1	0
1972	0	0	0	15	182	57	5	0	0
1973	0	0	0	38	218	62	3	0	0
1974	0	0	1	41	183	46	3	0	0
1975	0	0	12	106	266	92	4	1	0
1976	0	0	7	118	255	59	3	1	0
1977	0	0	0	35	262	90	5	0	0
1978	0	0	1	78	216	44	3	1	0
1979	0	0	1	117	273	72	4	1	0
1980	0	0	1	155	327	54	6	1	0
1981	0	0	11	302	480	55	7	0	0
1982	0	0	9	254	313	54	2	0	0
1983	0	0	10	275	500	96	3	0	0
1984	0	0	7	259	333	54	0	0	0
1985	0	0	13	222	343	45	5	0	0
1986	0	0	11	270	359	46	2	0	0
1987	0	1	16	222	337	40	0	0	0
1988	0	0	33	400	397	64	1	0	0
1989	0	1	184	801	385	73	5	0	0
1990	2	8	505	843	537	70	3	3	0
1991	1	14	459	838	344	44	0	0	0
1992	2	6	360	845	363	55	3	1	0
1993	8	18	2097	1214	409	82	2	0	0
1994	0	19	1998	1511	324	60	6	0	0
1995	0	25	957	1118	555	56	4	1	0
1996	3	2	527	716	509	61	7	0	0
1997	0	9	286	366	567	57	5	1	0
1998	0	234	1021	652	418	47	10	0	0
1999	0	132	911	959	476	44	3	0	0
2000	1	213	676	723	381	55	2	1	0
2001	0	166	742	1276	448	50	4	0	0
2002	0	248	1407	1280	485	75	8	0	0
2003	80	739	1197	1794	438	68	9	0	0
2004	41	483	1121	2765	636	71	4	0	0
2005	0	15	849	2861	763	80	5	0	0
2006	0	3	737	2771	706	96	6	1	0
2007	1	10	682	3306	737	96	6	0	0
2008	0	0	785	3314	670	101	11	0	0
2009	0	0	0	10	365	98	3	0	0
2010	0	0	1	8	531	105	3	0	0
2011	0	0	0	10	696	85	6	1	0
2012	12	0	0	2	583	90	3	0	0
2013	3	0	1	25	580	69	6	0	0
2014	0	0	0	28	803	78	5	0	0
2015	0	0	4	22	619	67	3	1	0
**Total**	**154**	**2347**	**17640**	**33010**	**20922**	**3388**	**216**	**18**	**1**

**Fig. 5:**
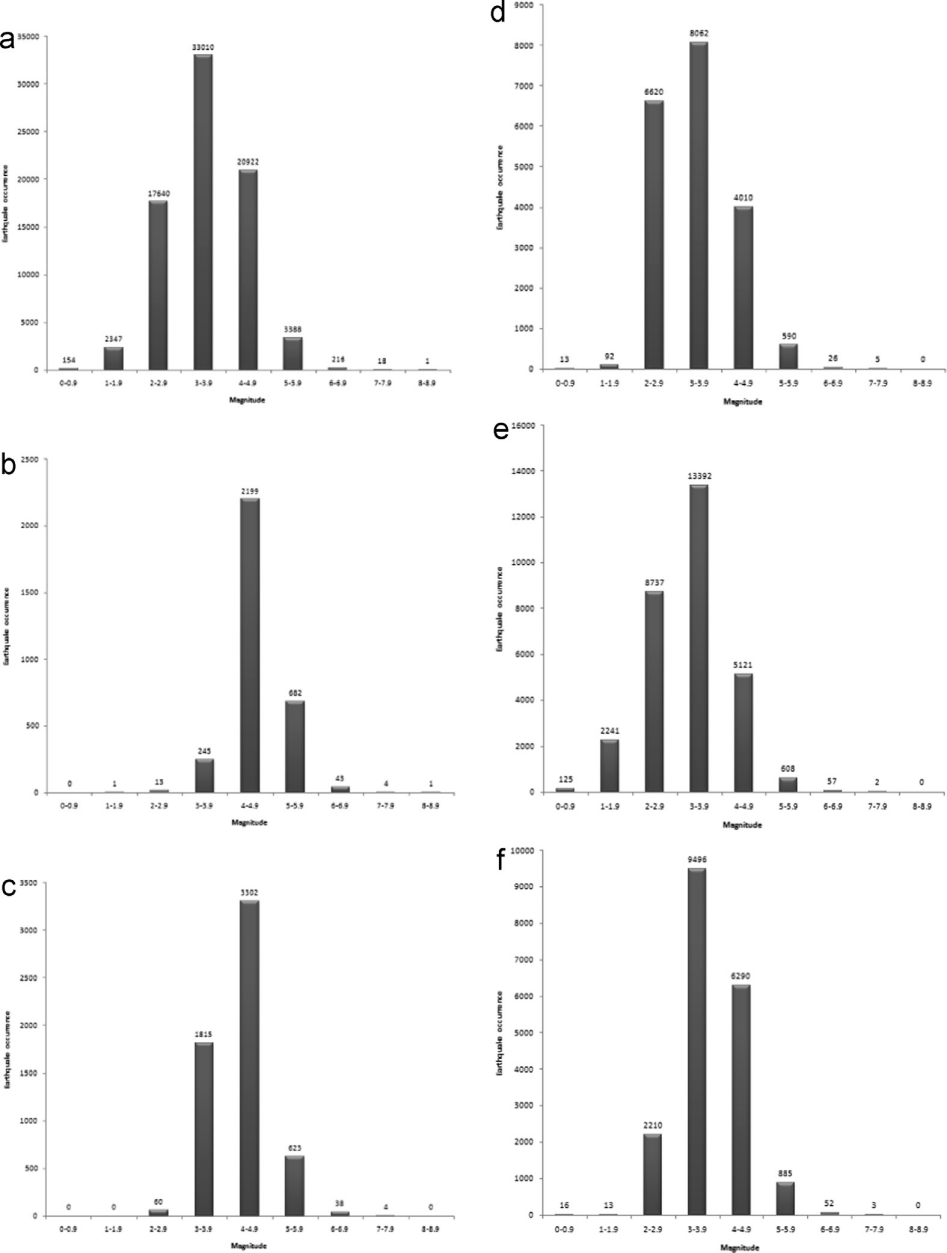
**a.** Earthquake occurrence – magnitude distribution from 1966 to 2015 **b.** Earthquake occurrence – magnitude distribution from 1966 to 1975 **c.** Earthquake occurrence – magnitude distribution from 1976 to 1985 **d.** Earthquake occurrence – magnitude distribution from 1986 to 1995 **e.** Earthquake occurrence – magnitude distribution from 1996 to 2005 **f.** Earthquake occurrence – magnitude distribution from 2006 to 2015.

**Fig. 6:**
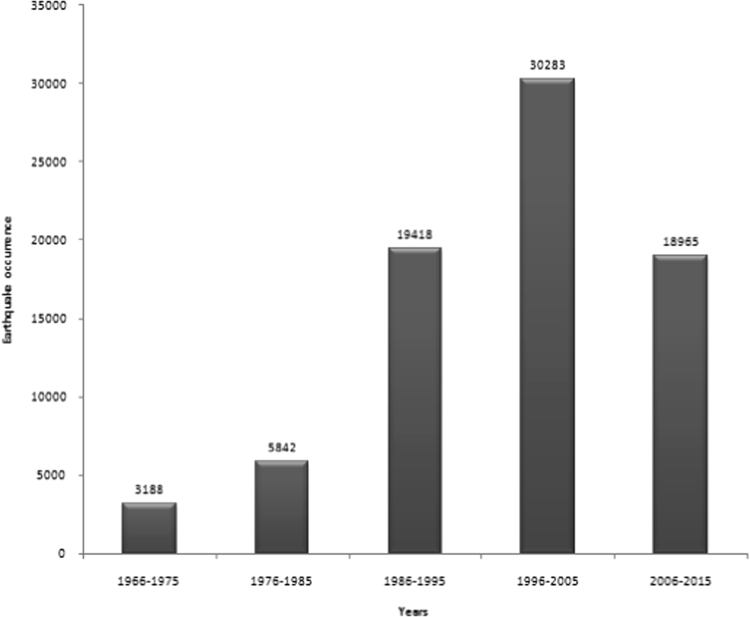
Decadal frequency distribution of earthquake occurrence in all the data sets.

The data sets were further explored by constructing the histograms of frequency of earthquake occurrence against the magnitude for the period of 50 years (1966–2015) (Fig. 5a), and 10-year interval: 1966–1975 (Fig. 5b), 1976–1985 (Fig. 5c), 1986–1995 (Fig. 5d), 1996–2005 (Fig. 5e), and 2006–2015 (Fig. 5f). The histogram of Fig. 5a (1966–2015) revealed that events of 3 ≤ *M* ≤ 3.9 earthquakes were most frequent, with the total events of 33010. In Fig. 5b and c (1966–1975 and 1976–1985), the most frequent magnitude is 4 ≤ *M* ≤ 4.9, with the total events of 2199 and 3302 respectively. However, Fig. 5d–f (1986–1995, 1996–2005, 2006–2015) showed that the most frequent magnitude is 3 ≤ *M* ≤ 3.9, with the total events of 8062, 13392, and 9496 respectively. The decadal frequency distribution of earthquake occurrence plotted in Fig. 6 showed that 1996–2005 is the most active decade of seismic events, with 30283 being the total earthquake occurrence in this decade.

The focal depths of the earthquake data sets were also analyzed. The focus of an earthquake is the actual point where the rocks break in the subsurface. The depths of the focus are categorized as shallow (0–70 km), intermediate (71–300 km), and deep depth (301–700 km below the earth surface) respectively [8]. Most earthquakes occurred at shallow depth, with total events of 76223, while deep depth had a total record of 61 times, with the magnitude ranging from 2 to 4.9. The focal depth distributions of earthquakes in the African-Arabian region during 1966–2015 are presented in Table 3.

**Table 3:** Magnitude – focal depth relationship of the earthquake occurrence.

**Magnitude range**	**Shallow depth (0**–**70** **km)**	**Intermediate depth (71**–**300** **km)**	**Deep depth (301**–**700** **km)**
0–0.9	154	0	0
1–1.9	2343	4	0
2–2.9	17,473	166	1
3–3.9	32,510	478	22
4–4.9	20,232	699	38
5–5.9	3281	60	0
6–6.9	211	5	0
7–7.9	18	0	0
8–8.9	1	0	0
Total	76223	1412	61

The frequency-magnitude distributions of earthquake occurrences for the period of 50 years (1966–2015) and 10-year interval covering the entire investigated period were produced through the GR relation (Eq. (1)). This was achieved by plotting the graph of cumulative number of earthquakes against their respective magnitudes. The graphs were fitted, with a linear fitting. The equation from the linear fitting represents the GR relation, where the slope of the graph stands for the *b*-value. However, the *a*-value (seismicity level) was estimated by substituting the known parameters into the GR equation. The magnitude of completeness (*M*_C_) of the earthquake catalogue was also determined for each period. The *M*_C_ is referred to as the threshold magnitude which is the magnitude above which all earthquakes were recorded.

**Fig. 7:**
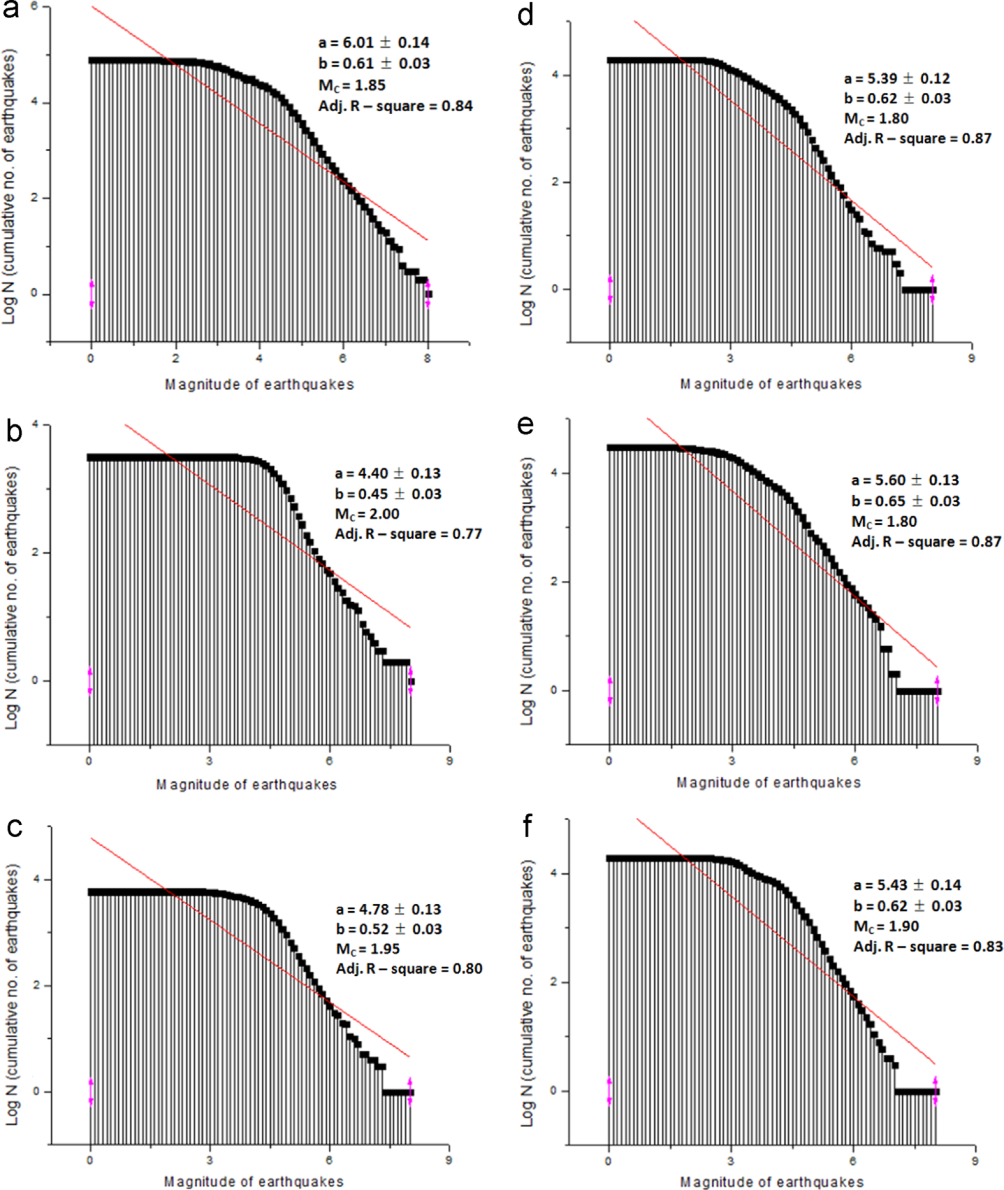
**a.** Log N – magnitude distribution from 1966 to 2015 **b.** Log N – magnitude distribution from 1966 to 1975 **c.** Log N – magnitude distribution from 1976 to 1985 **d.** Log N – magnitude distribution from 1986 to 1995 **e.** Log N – magnitude distribution from 1996 to 2005 **f.** Log N – magnitude distribution from 2006 to 2015.

The GR plots for the period of 1966–2015, 1966–1975, 1976–1985, 1986–1995, 1996–2005, 2006–2015 were presented as Fig. 7a–f, with the *a*-value, *b*-value, *M*_C_ value and Adjusted R-square (Adj. R-square) value of each analysis being displayed on each graph. The Adj. R-square is a corrected goodness of fit (model accuracy). It is calculated by dividing the residual mean square of error by the total mean square error. The complete data sets during the period of 1966–2015 revealed that the *b*-value of the study area is 0.61, *a*-value is 6.01, *M*_C_ is 1.85, and Adj. R-square is 0.84 respectively. The analysis across the five (5) decades showed that the *b*-value fluctuates from 0.45 (during the first decade) to 0.65 (during the fourth decade), *a*-value from 4.40 (during the first decade) to 5.60 (during the fourth decade). The *M*_C_ varied from 1.80 to 2.00.

Statistical analysis involving the Analysis of Variance (ANOVA) was carried out on the magnitude of the earthquakes. This analysis is used to determine the difference in the mean of sets of data or groups. The ANOVA uses *F*-tests to statistically analyze or test the equality of means. The test was named after Sir Ronald Fisher. The *F*-test is the ratio of two variances (measure of dispersion), being the square of standard deviation. The ANOVA results of the data sets covering the period of 1966–2015, and five decades (1966–1975, 1976–1985, 1986–1995, 1996–2005, and 2006–2015) are presented in Table 4. The ANOVA results showed that *F*-values recorded for the periods of 1966–2015 (being the whole coverage year for the analysis) is 430.73, 1966–1975 is 267.62, 1976–1985 is 322.91, 1986–1995 is 527.75, 1996–2005 is 535.97, and 2006–2015 is 405.09 respectively.

**Table 4:** ANOVA results and summary of the parameters from frequency-magnitude curves.

Year	Source	DF	Sum of squares	Mean of square	*F*-value	Prob > *F*	*a*-value	*b*-value	*M*_C_	Adj. R-square
1966–2015	Model	1	165.30	165.30	430.73	0	6.01	0.61	1.85	0.84
	Error	79	30.32	0.38						
	Total	80	195.62							
										
1966–1975	Model	1	87.91	87.91	267.62	0	4.40	0.45	2.00	0.77
	Error	79	25.95	0.33						
	Total	80	113.86							
										
1976–1985	Model	1	118.00	118.00	322.91	0	4.78	0.52	1.95	0.80
	Error	79	28.87	0.37						
	Total	80	146.86							
										
1986–1995	Model	1	171.46	171.46	527.75	0	5.39	0.62	1.80	0.87
	Error	79	25.67	0.32						
	Total	80	197.13							
										
1996–2005	Model	1	185.04	185.04	535.97	0	5.60	0.65	1.80	0.87
	Error	79	27.27	0.35						
	Total	80	212.31							
										
2006–2015	Model	1	168.18	168.18	405.09	0	5.43	0.62	1.90	0.83
	Error	79	32.80	0.42						
	Total	80	200.98							

**Fig. 8:**
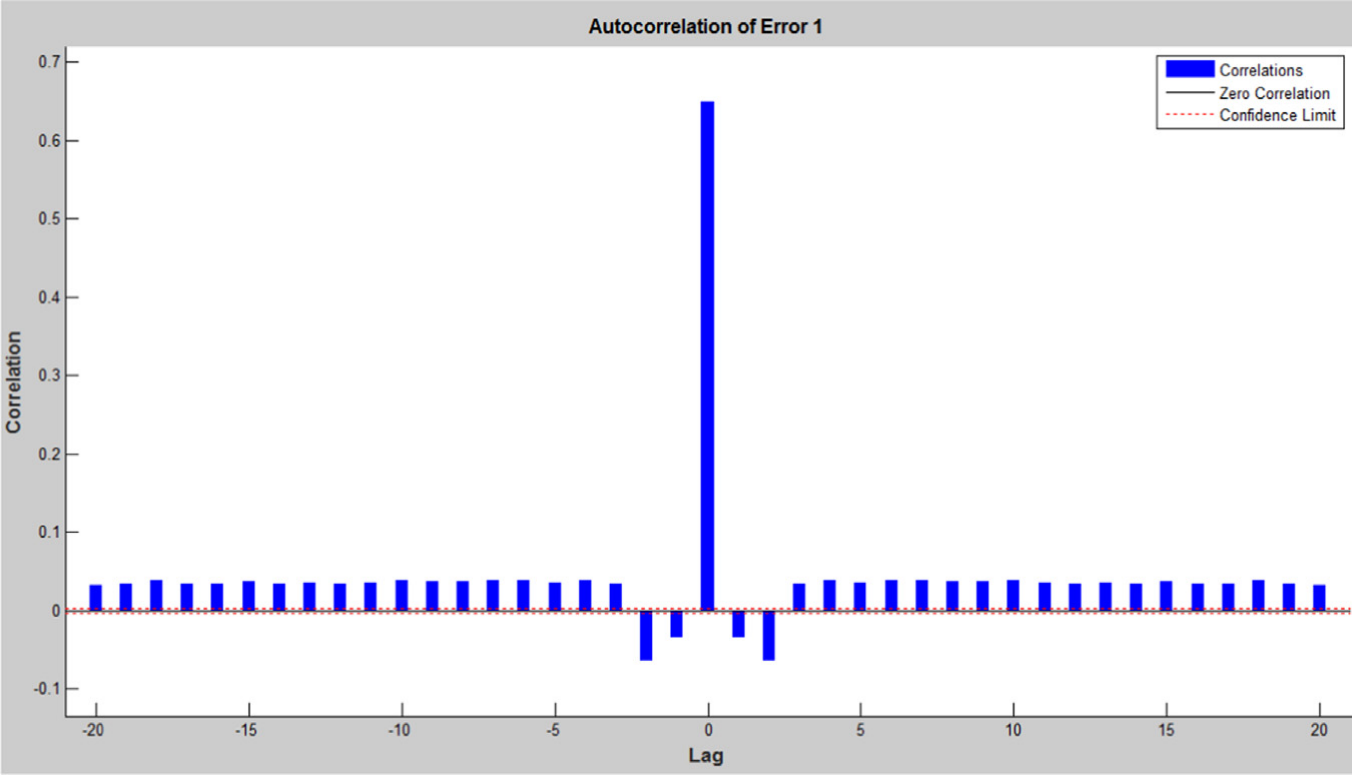
Neural network training of error autocorrelation, Epoch 291.

**Fig. 9:**
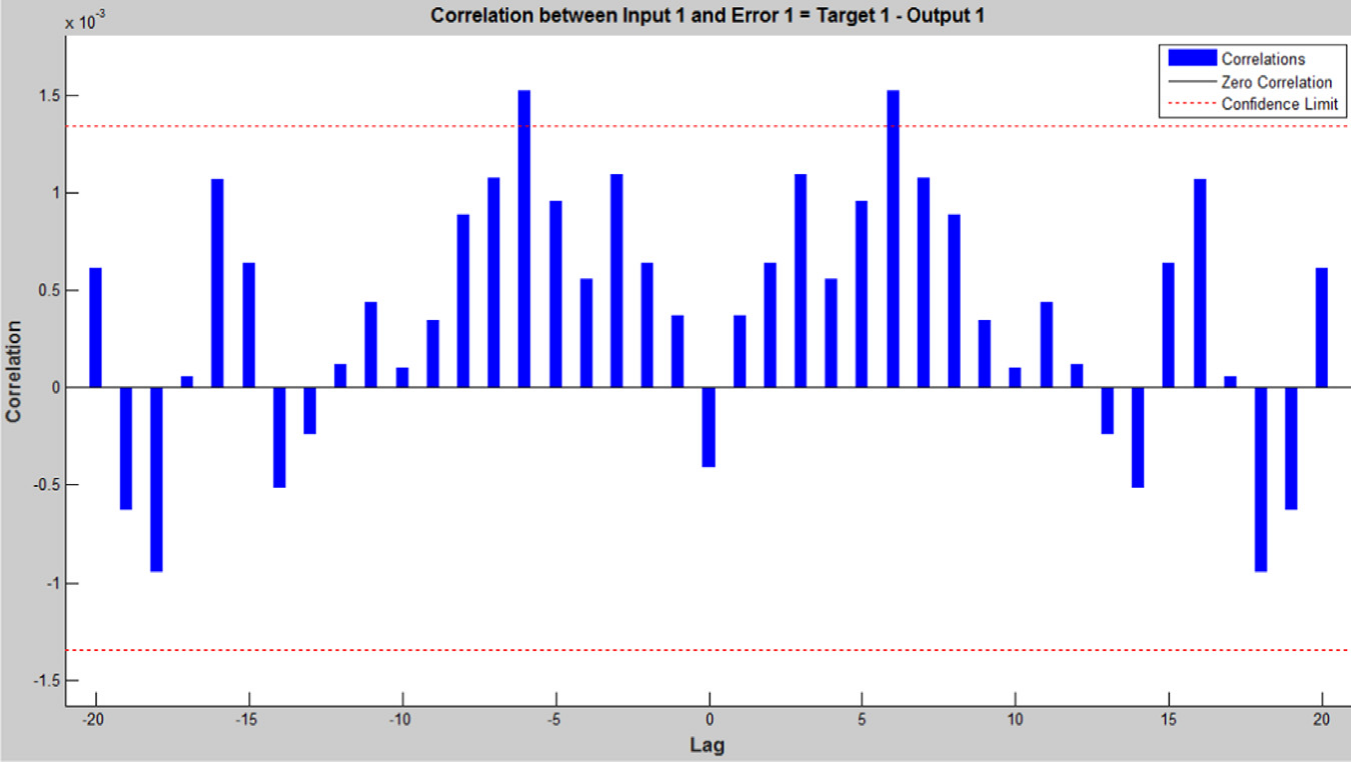
Neural network training of input-error cross-correlation, Epoch 291.

**Fig. 10:**
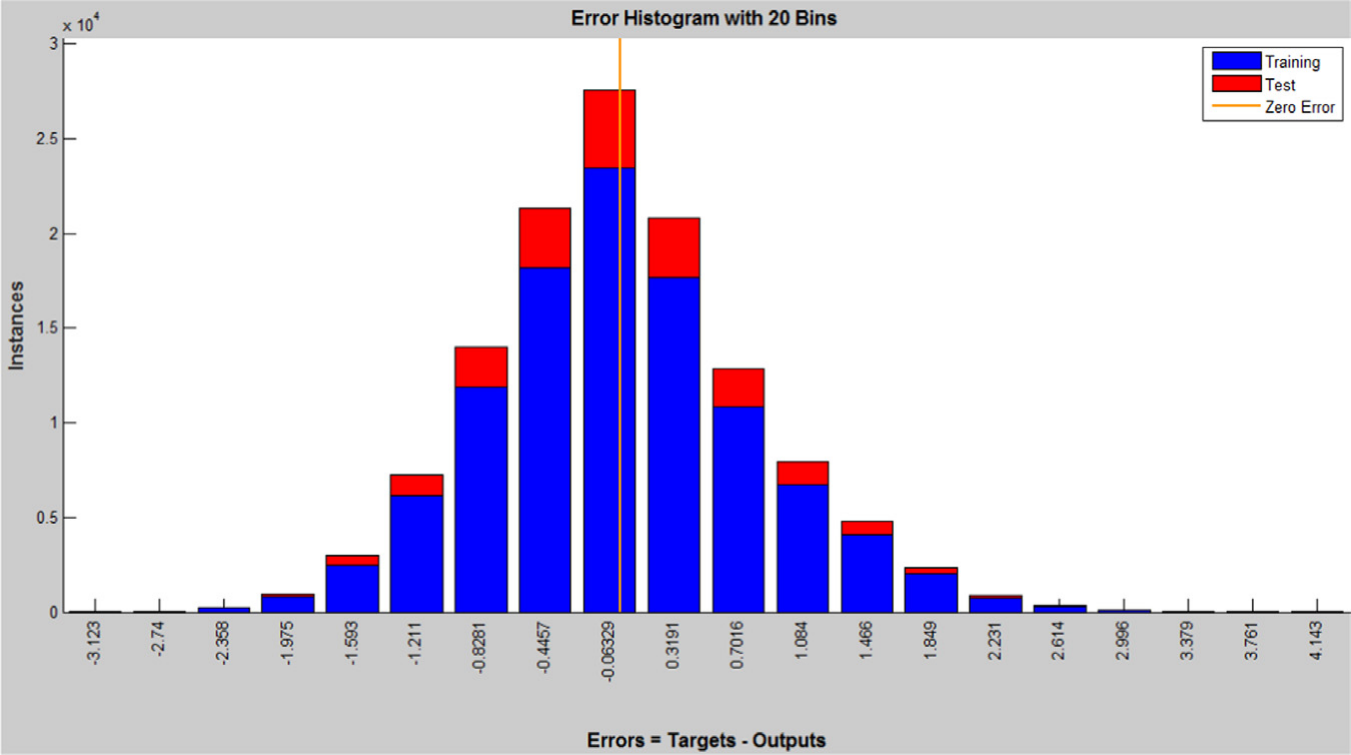
Neural network training of error histogram with 20 bins, Epoch 291.

**Fig. 11:**
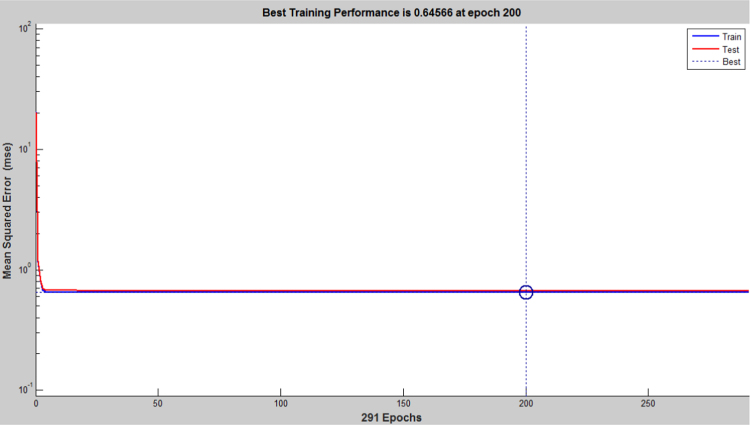
Neural network training of performance 0.64566 at epoch 200 and iteration 20.

**Fig. 12:**
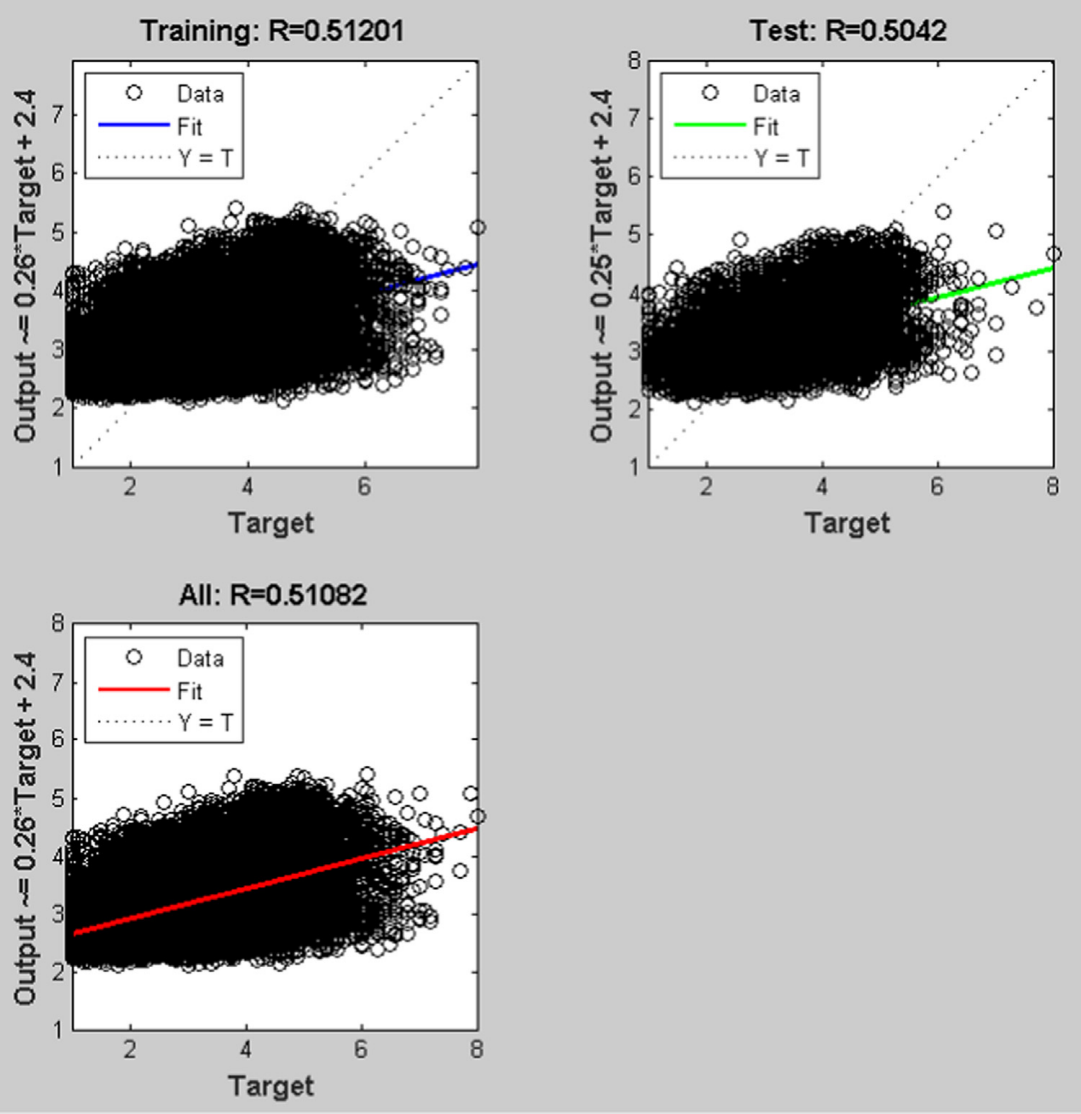
Regression analysis of the dataset with R-square of > 0.5 in all cases.

**Fig. 13:**
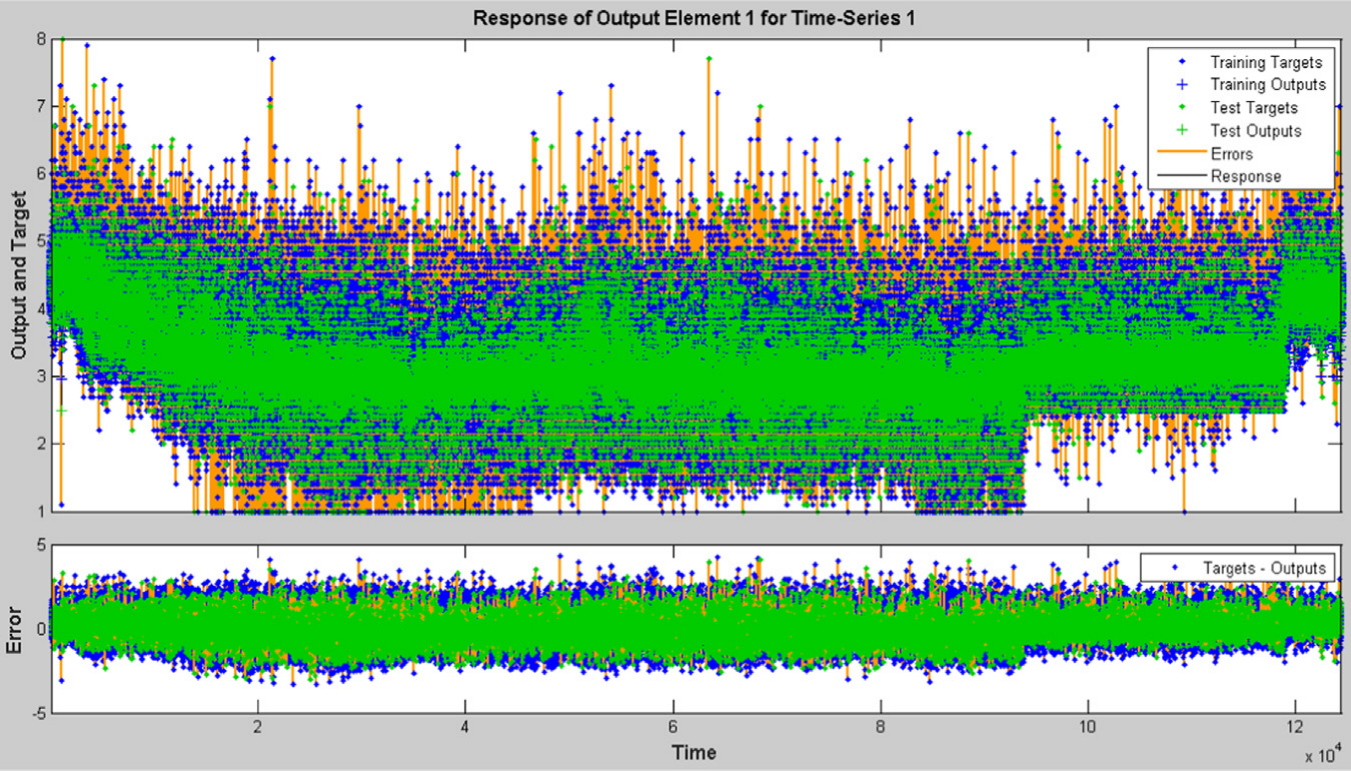
Time series response for the dataset, Epoch 291.

The earthquake forecasting approach from historic seismic data is prevalent nowadays [11], which was further used to evaluate the earthquake data sets. This technique employing a dynamic neural network was further used to explore the earthquake data sets (the neural network function and the source code were attached as Supplementary files). This approach is good at time series prediction. With the total magnitude of 77, 696, graphical user interfaces and command-line functions were used to produce the codes and figures for earthquake prediction in this article. The dataset was selected and the problem to solve was defined in the MATLAB. The network was trained in order to fit a time series data set. The beauty of using neural network time series tool (ntstool) is that it is capable to solve three problems differently. The first task which was adopted in this analysis is to predict future values from previous values *y*(*t*) and past values from a second time series *x*(*t*) using a nonlinear autoregressive with exogenous input (NARX). The second task is to only predict future values from the past values of such time series using a nonlinear autoregressive (NAR) input. The third task is to predict future values from the previous values without having knowledge of previous values using input–output model instead of NARX. The magnitudes of the earthquake were imported, validated and tested such that 70% of the data were used for the training while 15% each will be used for validation and testing of the dataset. The graphical interfaces of the treated data were presented from Fig. 8, Fig. 9, Fig. 10, Fig. 11, Fig. 12, Fig. 13.

The qualitative and quantitative approach employed in this article can be beneficial in the study of stability of the African lithospheric plates. The data sets can be reexamined to estimate the time scale dependence of earthquake parameters in subregions of Africa and/or Middle East. The approach presented in this article can provide insights to researchers on further explorations of aseismic zones being affected by tremors in Africa such as Nigeria [21], [22], [23]. However, despite the challenges being faced in earthquake predictions, it has been noted that the beauty of neural network is to predict the next major seismic event [11]. Analysis of the dataset presented in this article can be used to forecast the earthquake occurrence in African – Western Asia region. If accurate forecast is achieved in this region, it would be beneficial for the masses since danger of loss of lives and properties would be reduced.

## Footnotes

Appendix A Supplementary data associated with this article can be found in the online version at doi:10.1016/j.dib.2018.01.049.

## References

[1] Adagunodo T.A., Sunmonu L.A. (2015). Earthquake: a terrifying of all natural phenomena. J. Adv. Biol. Basic Res..

[2] Florido E., Aznarte J.L., Morales-Estebam A., Martinez-Alvarez F. (2016). Earthquake magnitude prediction based on artificial neural networks: a survey. Croat. Oper. Res. Rev..

[3] Moustra M., Avraamides M., Christodoulou C. (2011). Artificial neural networks for earthquake prediction using time series magnitude data or seismic electric signals. Expert Syst. Appl..

[4] Marzocchi W., Sandri L. (2003). A review and new insights on the estimation of the b-value and its uncertainty. Ann. Geophys..

[5] Hammed O.S., Awoyemi M.O., Igboama W.N., Badmus G.O., Essien U.C. (2016). Pattern of seismicity associated with the African lithospheric plate. Phys. Sci. Int. J..

[6] Gutenberg B., Richter C. (1956). Magnitude and energy of earthquakes. Ann. Geofis..

[7] Hammed O.S., Awoyemi M.O., Badmus G.O., Sanni O.O. (2016). Interdependence an variations of earthquake parameters on African lithospehric plate using Guterberg and Richter relations. J. Geogr. Environ. Earth Sci. Int..

[8] Hammed O.S., Popoola O.I., Adetoyinbo A.A., Awoyemi M.O., Badmus G.O., Ohwo O.B. (2013). Focsl depth, magnitude, and frequency distribution of earthquakes along oceanic trenches. Earthq. Sci..

[9] Alabi A.A., Akinyemi O.D., Adewale A. (2013). Seismicity pattern in southern Africa from 1986 to 2009. Earth Sci. Res..

[10] Grant R.A., Haliday T. (2010). Predicting the unpredictable; evidence of pre-seismic anticipatory behaviour in the common toad. J. Zool..

[11] Morales-Esteban A., Martinez-Alvarez F., Troncoso A., Justo J.L., Rubio-Escudero C. (2010). Pattern recognition to forecast seismic time series. Expert. Syst. Appl..

[12] Adagunodo T.A., Sunmonu L.A., Adabanija M.A., Suleiman E.A., Odetunmibi O.A. (2017). Geoexploration of Radioelement’s datasets in a flood plain of Crystalline bedrock. Data Brief.

[13] Bishop S.A., Owoloko E.A., Okagbue H.I., Oguntunde P.E., Odetunmibi O.A., Opanuga A.A. (2017). Survey datasets on the externalizing behaviours of primary school pupils and secondary school students in some selected schools in Ogun State, Nigeira. Data Brief.

[14] Adejumo A.O., Suleiman E.A., Okagbue H.I. (2017). Exploration of solar radiation data from three geo-political zones in Nigeria. Data Brief.

[15] Burke K. (1996). The African plate: the 24th du Toit Memorial Lecture. Afr. J. Geol..

[16] Zitellini N., Gracia E., Matias L., Terrinha P., Abreu M.A., DeAlteriis G., Henriet J.P., Danobeitia J.J., Masson D.G., Mulder T., Ramella R., Somoza L., Diez S. (2009). The quest for the Africa-Eurasia plate boundary west of the straight of Gibraltar. Earth Planet. Sci. Lett..

[17] McClusky S., Reilinger R., Mahmoud S., Ben Sari D., Tealeb A. (2003). GPS constraints on Africa (Nubia) and Arabia plate motions. Geophys. J. Int..

[18] Logatchev N.A., Beloussov V.V., Milanovsky E.E. (1972). East African Rift Development. Dev. Geotecton..

[19] Plummer P.S., Belle E.R. (1995). Mesozoic tectono-stratigraphic evolution of the Seychelles Microcontinent. Sediment. Geol..

[20] Schluter T., Trauth M.H. (2008). Geological Atlas of Africa: with notes on Stratigraphy, Tectonics, Economic Geology, Geohazards, Geosites and Geoscientific Education of each Country.

[21] Tsalha M.S., Lar U.A., Yakubu T.A., Kadiri U.A., Duncan D. (2015). The review of the Historical and recent seismic activity in Nigeria. IOSR J. Appl. Geol. Geophys..

[22] Akpan O.U., Yakubu T.A. (2010). A review of earthquake occurrences and observations in Nigeria. Earthq. Sci..

[23] P.O. Awoyera, B.U. Ngene, G.A. Adeyemi, P.A. Aderonmu, Mitigating Ground Shaking Construction Activities in Coastal Cities of Nigeria: An Earthquake Preventive Measure. In: Earthquakes: Monitoring Technology, Disaster Management and Impact Assessment. Nova Science Publishers, Inc., United States of America. ISBN 978-1536103427.

